# An Epidermoid Cyst in the Neck Mimicking a Thyroglossal Duct Cyst: A Case Report

**DOI:** 10.7759/cureus.77366

**Published:** 2025-01-13

**Authors:** Yash A Gandhi, Ajeet K Khilnani, Narendra Hirani, Hetal Joshi, Nisarg Desai

**Affiliations:** 1 Otolaryngology and Head and Neck Surgery, Gujarat Adani Institute of Medical Sciences, Bhuj, IND

**Keywords:** benign neck swelling, dermoid, epidermoid cyst, neck swelling, thyroglossal duct cyst

## Abstract

Anterior neck masses, especially congenital neck masses are one of the most common causes of visits to an ENT specialist. Commonly encountered masses include thyroid swellings, thyroglossal duct cyst, lymph nodes, dermoid/epidermoid cysts, brachial cleft cysts, and lymphatic malformations. Hence a thorough clinical history and examination make an important part of evaluation supported by radiological and pathological correlation. Here we report a case of a 44-year-old male patient presenting with anterior neck swelling which was initially diagnosed as a thyroglossal cyst but on histopathological examination turned out to be an epidermoid cyst.

## Introduction

Swelling in the anterior neck is one of the most common causes of visits to an ENT specialist. The common swellings of the anterior neck are thyroid swellings, thyroglossal cysts, pre-tracheal lymph nodes, and dermoid [1]. Most of the time diagnosis of neck swelling is established by clinical, radiological, and pathological correlation. However, sometimes there can be difficulty in diagnosis when the swelling is either present at an unusual location or presents with unusual features [2]. Epidermoid cyst in the neck is usually situated in subcutaneous tissue, and superficial to strap muscles, near the suprasternal notch with a microscopic picture showing fat or calcification [3]. Here we report a case of a 44-year-old male patient with anterior neck swelling, who was operated on with a diagnosis of a thyroglossal cyst which on histopathological examination (HPE) turned out to be an epidermoid cyst. The patient was asymptomatic at one-year follow-up.

## Case presentation

A 44-year-old male patient, a resident of Kachchh district, Gujarat, India, and working as a laborer, came to the ENT outpatient department with a complaint of midline neck swelling for 10 years. The swelling was initially small in size but gradually and painlessly increased to its present size. There were no complaints of fever, pain, any other swelling, difficulty in swallowing, difficulty in breathing, earache, or nasal discharge.

**Figure 1:**
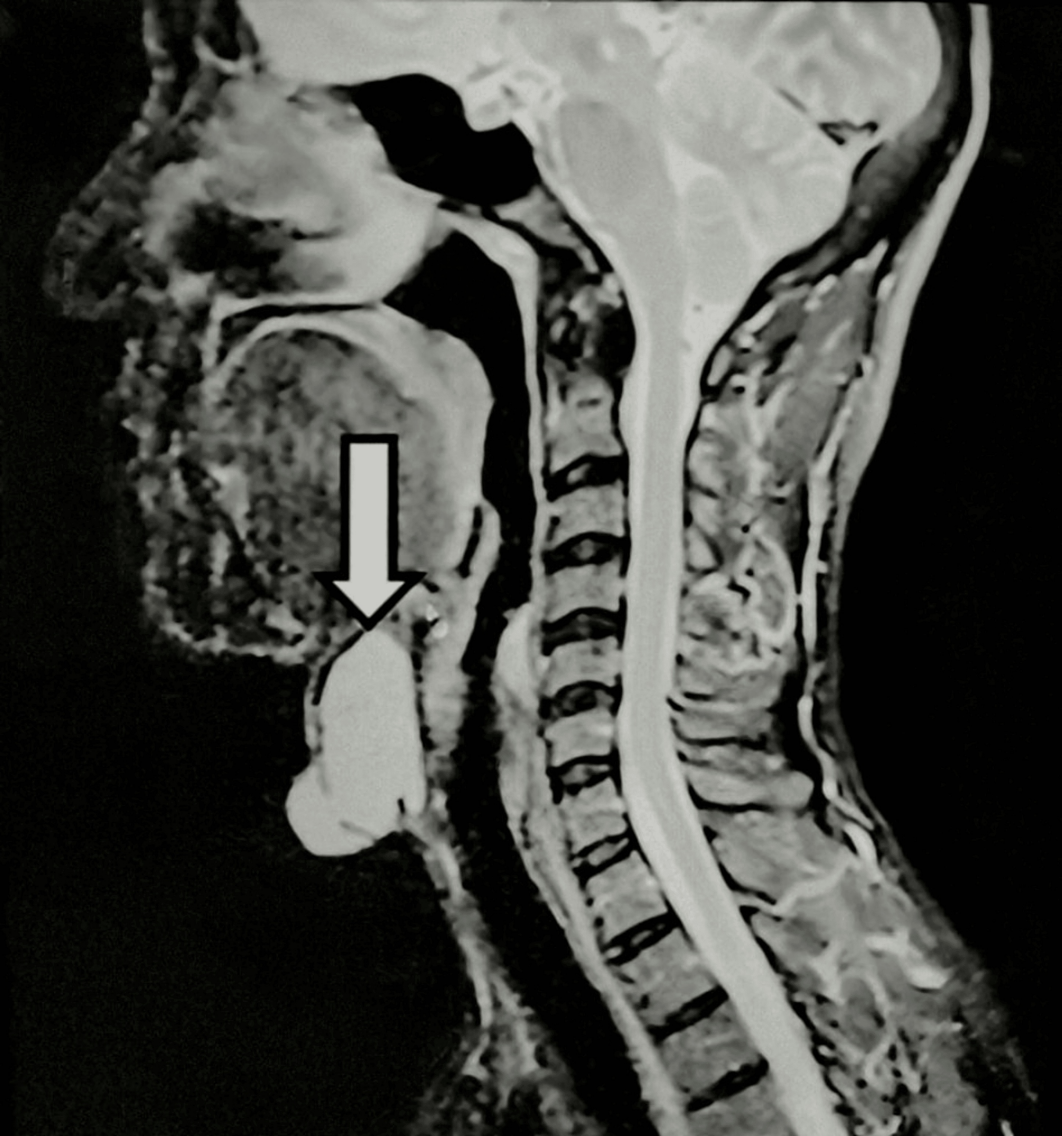
MRI neck (sagittal view) showing a well-defined cystic lesion in the midline of the neck (white arrow) that is hyperintense on T1- and T2-weighted images. There is no evidence of communication with the thyroid gland

On inspection, there was a single spherical swelling of 2 cm × 2 cm in size, with clearly defined edges and a smooth surface. The swelling moved with deglutition but did not move with protrusion of the tongue. There were no visible pulsations, impulse on coughing was absent, and no pigmentation of overlying skin was seen. On palpation, it was a non-tender, 2 cm × 2 cm × 1 cm cystic spherical swelling. A clinical diagnosis of benign thyroid swelling was made, and ultrasound (USG) and cytology (fine needle aspiration cytology (FNAC)) were advised. USG showed an exophytic lobulated cystic lesion, infra-hyoid in location and separate from the thyroid gland, with fine internal echoes with a possible diagnosis of thyroglossal cyst. Cytology also reported a benign cystic lesion with a possibility of a thyroglossal cyst. To further assist the diagnosis, an MRI was done, which showed a well-defined cystic lesion in the midline suprahyoid region with no evidence of communication with the thyroid gland (Figure 1). Based on the clinical, radiological, and cytological evaluation, a diagnosis of thyroglossal cyst was made, and Sistrunk surgery was planned.

**Figure 2:**
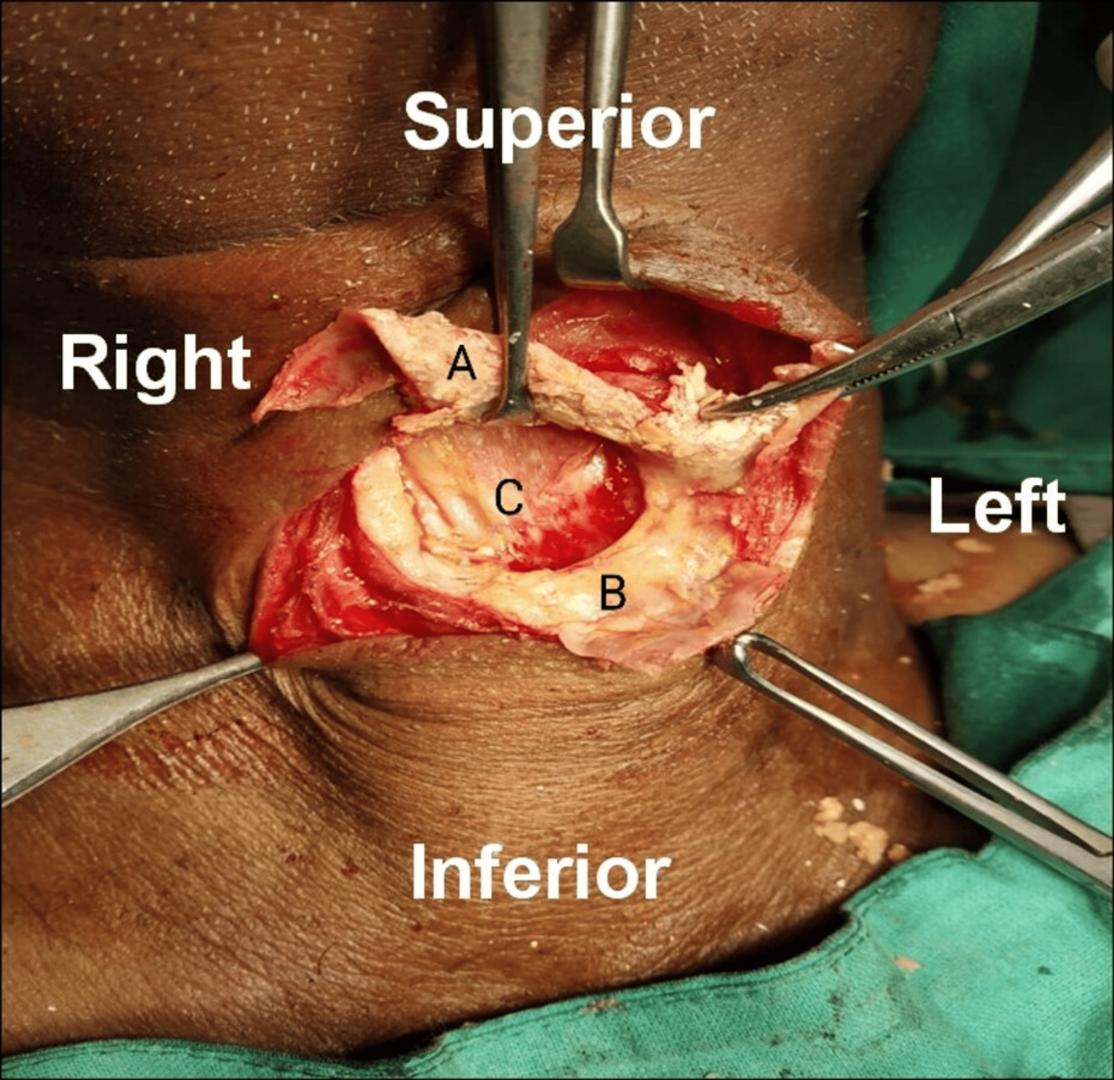
Intraoperative image showing the anterior cyst wall (A), thick peripheral rim (B), and absent posterior wall (C). Strap muscles can be seen at the base of the swelling

During surgery, the cyst was exposed and had a small opening in the anterior wall with yellowish, cheesy material coming out. The posterior wall of the cyst could not be dissected from the underlying strap muscles (Figure 2).

**Figure 3:**
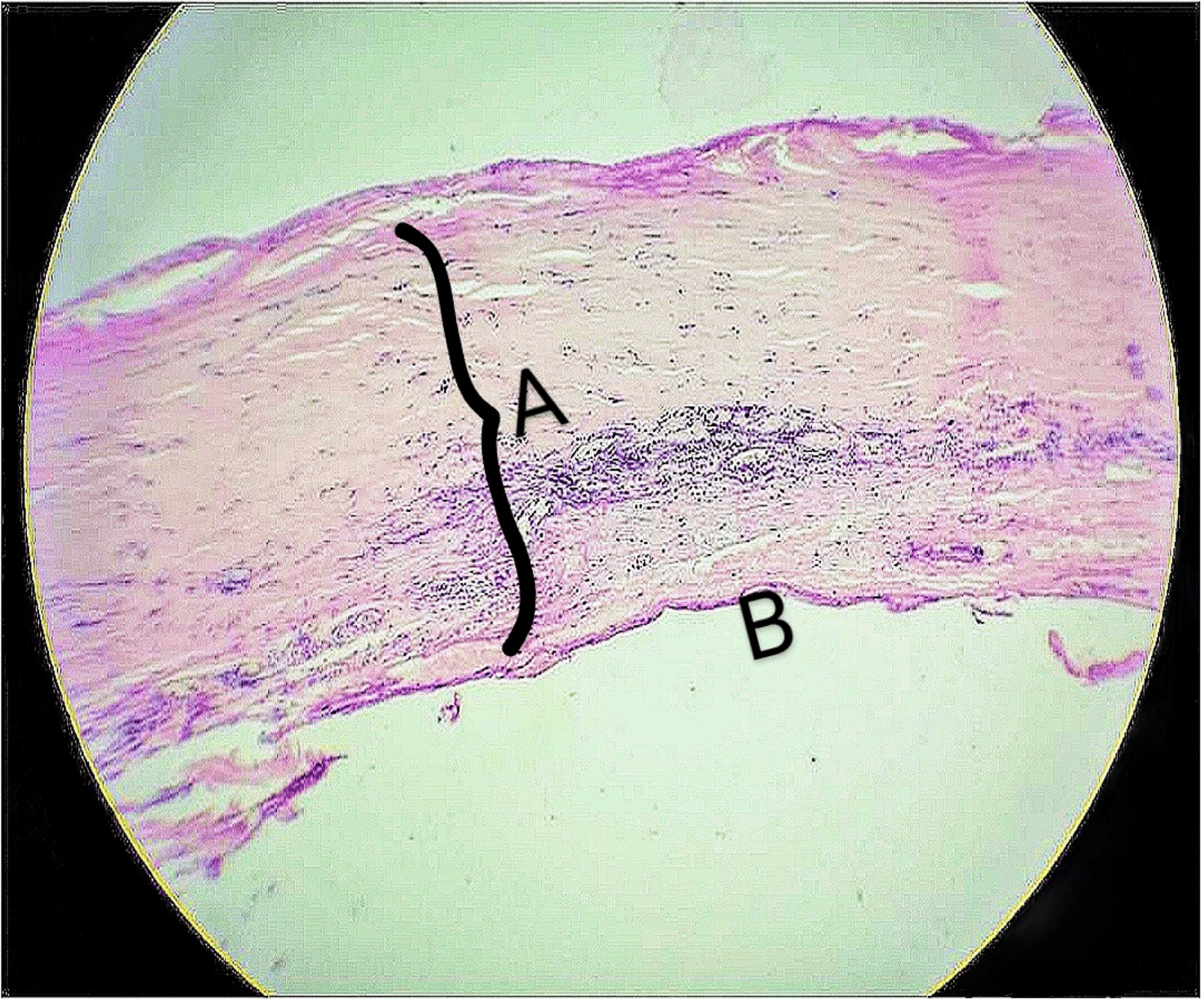
H&E scanner view (× 50) showing cyst wall with fibro-collagenous tissue and chronic inflammatory cells (A). The cyst wall is lined by flattened epithelium (B)

Except for the thick rim, which could not be excised due to adherence with underlying structures, the cyst was excised and sent for HPE. There was no communication of the cyst with the hyoid bone. The final histopathological diagnosis was a benign epithelial cyst (epidermoid cyst) (Figure 3). The patient was followed up for one year, and there was no recurrence noted.

## Discussion

There are many differential diagnoses for anterior neck swellings, the most common being thyroid swelling, thyroglossal duct cyst, lymph node enlargement (cervical lymphadenopathy) due to various causes, including reactive, metastatic, as well as Koch’s lymphadenopathy, dermoid/epidermoid cysts, lymphomas, abscesses, etc. Thyroid swellings, as well as thyroglossal cysts, move with deglutition but are differentiated as thyroglossal cysts also move with protrusion of the tongue, whereas thyroid swellings don’t. Dermoid and epidermoid cysts are a part of the spectrum of congenital and acquired cystic malformations that share the common characteristic of having a squamous epithelial lining. About 7% are present in the head and neck region [4]. They can present as cystic spaces lined by simple squamous epithelium (epidermoid cysts), may contain skin adnexa (true dermoid cysts), or contain tissues of all three germ layers (teratoid cysts). An epidermoid cyst is commonly situated in the subcutaneous region, superficial to strap muscles, mostly near the suprasternal notch. Whereas, on the contrary, thyroglossal duct cysts are specifically present in a deeper location, lodged within strap musculature within close proximity to the hyoid bone [5]. Epidermoid cysts show diffusion restriction on MRI [6]. In our case, it was an epithelial cyst with no adnexal structures. Epidermoid cysts are present in early life, generally during infancy [7]. However, in our case, the patient first noticed the swelling at 34 years of age. The anterior neck swellings are managed mainly surgically. Preoperative radiology, as well as FNAC, guides us in diagnosing the swellings. In our case, USG of the local part as well as cytology done pre-operatively were suggestive of a thyroglossal duct cyst, which was seconded by an MRI of the neck, and the post-operative histopathology was suggestive of an epidermoid cyst.

## Conclusions

This is a case of an epidermoid cyst of the neck mimicking a thyroglossal cyst. There should always be a discussion between radiologists, pathologists, and ENT surgeons whenever there is a doubt in the diagnosis of neck swelling. A sharp understanding of the differentiating features of cystic neck masses is important in light of different clinical implications; readiness for intraoperative surprises is essential.
